# On the Treatment of Tape-Worm by the Oil of Pumpkin-Seeds

**Published:** 1853-11

**Authors:** 


*On the Treatment of Tape-worm by the Oil of Pumpkin-seeds.* By Henry S. Patterson, M. D., Professor of Materia Medica in the Medical Department of Pennsylvania College.

In the Medical Examiner for October, 1852, I reported a case of radical cure of tænia by the use of an emulsion of pumpkin-seeds, after the ol. terebinth, and even kousso had signally failed. Several other cases have been reported, both before and since mine, all going to establish the efficacy of this new remedy. Should it prove as generally successful in expelling the worm as the cases indicate, it will become a valuable accession to our means of treatment in a troublesome and often obstinate affection.

The seeds of the common pumpkin, (*cucurbita pepo,*) consist of a leathery white envelope, inclosing an oily albumen of a slightly greenish tinge. They are inodorous and have a sweetish, mucilaginous taste. Rubbed up with warm water or milk, and sweetened, they form a very pleasant emulsion; and this is the way in which they have generally been administered. They abound in fixed oil, which is readily yielded on expression, and appears to be the only constituent of any importance. Conceiving this oil to be the anthelmintic principle, I determined to use it in the first case of tænia I should encounter. A quantity was obtained, by cold expression, by our accomplished pharmaceutist, Mr. Frederick L. John, of Race street. From 4lbs. of the seeds he procured ℥xiv. of oil, but he has no doubt that, if the operation were conducted on a larger scale and more carefully, the yield would be from thirty to forty per cent. The oil is clear, transparent, of a light brownish green color, with a slight oily odor, and a perfectly bland taste, like that of the oil of sweet almonds. It has now been kept some ten months, (in well-stoppered bottles,) and is perfectly sweet and bland.

No case of tænia has occurred in my own practice, but in May last I learned from Mr. John C. Lyons, an intelligent member of the medical class of Pennsylvania College, that a poor woman in his neighborhood, (Kensington,) labored under the disease, and had asked his advice. I requested him to use the pumpkin-seed oil, which he did, and with the happiest result. Causing her to fast rigidly for twenty-four hours, he gave her ℥ss. of the oil in the morning, and in about two hours ℥ss. more. This produced a slight disposition to looseness of the bowels. In two hours after the last dose, ℥i. of castor-oil was given, and purged freely, bringing away a considerable quantity of the worm. From that period until the present, (September,) she has remained entirely free from every symptom of verminous irritation, and there can be no doubt that the worm was altogether destroyed.

Tænia is of so rare occurrence with us, that no individual practitioner sees enough of it to enable him alone fairly to test any medicine. I therefore beg leave to call the attention of my medical brethren to a remedy readily obtained, cheap and pleasant, and which I believe will be found quite efficient.—*Philadelphia Med. Examiner.*

